# Phenotypic and Genotypic Characterization of Candida parapsilosis complex isolates from a Lebanese Hospital

**DOI:** 10.21203/rs.3.rs-4169036/v1

**Published:** 2024-05-30

**Authors:** Reine El Hady, Nour Fattouh, Marc Finianos, Ibrahim Bitar, Rola Husni, Roy khalaf

**Affiliations:** Lebanese American University - Byblos Campus; Saint George University of Beirut; Charles University: Univerzita Karlova; Charles University: Univerzita Karlova; Lebanese American University School of Medicine; Lebanese American University - Byblos Campus

**Keywords:** Candida parapsilosis, Fluconazole, Pathogenicity, Resistance, Clonality

## Abstract

The opportunistic fungal pathogen *Candida parapsilosis* is a major causative agent of candidiasis leading to death in immunocompromised individuals. Azoles are the first line of defense in treatment by inhibiting *ERG11,* involved in the synthesis of ergosterol, the main sterol fungal sterol. Resistance to azoles is on the increase worldwide including in Lebanon. The purpose of this study is to characterize nine hospital isolates labeled as *C. parapsilosis*: four resistant and five sensitive to fluconazole. Phenotypic characterization was achieved through a battery of tests that target pathogenicity attributes such as virulence, biofilm formation, stress resistance, and ergosterol content. Genotypic analysis was done through whole genome sequencing to mutations in key virulence and resistance genes. Phylogenetic comparison was performed to determine strain relatedness and clonality. Genomic data and phylogenetic analysis revealed that three of the nine *C. parapsilosis* isolates were misidentified; two as *C. orthopsilosis* and *C. metapsilosis* belonging to the *C. parapsilosis* complex, while the third was *C. albicans*. Moreover, several known and novel mutations in key drug resistance and virulence genes were identified such as *ERG11, ERG3, ERG6, CDR1,* and *FAS2*. Phylogenetic analysis revealed a high degree of relatedness and clonality within our *C. parapsilosis* isolates. Our results showed that resistant isolates had no increased ergosterol content, no statistically significant difference in virulence, but exhibited an increase in biofilm content compared to the sensitive isolates. In conclusion, our study, the first of its kind in Lebanon, suggests several mechanisms of antifungal drug resistance in *C. parapsilosis* hospital isolates.

## Introduction

Infections brought on by invasive opportunistic fungal pathogens belonging to the *Candida* species have become much more common throughout the last 30 years. Up to 50% of all fatalities from fungal diseases can be attributed to invasive fungal infections, which are often linked to high rates of severe sickness [1]. *C. parapsilosis* presents a particular risk to newborns, recipients of organ transplants, and people on parenteral nutrition, and it belongs to the *Candida* clade that is characterized by the unique translation of the CUG codons as serine instead of leucine. Although *C. parapsilosis* infections have lower mortality and morbidity rates than *C. albicans,* several clinical isolates of *C. parapsilosis* have been reported to be less susceptible to echinocandins and resistant to azole treatment in some regions [2]. The *C. parapsilosis* complex is composed of three members: *C. parapsilosis, C. orthopsilosis* and *C. metapsilosis*. Before recent advances in genomics, they were indistinguishable and considered as one species, *C. parapsilosis,* up until 2005 [3]. Even nowadays, distinguishing between *C. parapsilosis, C. orthopsilosis* and *C. metapsilosis* is not possible through commercial systems, but only through DNA sequencing. Very few studies have investigated the virulence of *C. parapsilosis,* and even fewer that of *C. orthopsilosis* and *C. metapsilosis*. To date, only a few studies have shown that *C. orthopsilosis* and *C. parapsilosis* exhibit similar virulence and adhesion properties, while *C. metapsilosis* is generally considered the least virulent of the three species [4]. *C. metapsilosis* is also the least commonly isolated, causing between 0.6% and 6.9% of invasive candidiasis cases [5]. *C. parapsilosis* exhibits an oval, cylindrical round shape; it appears as white and creamy on potato dextrose agar with a shiny smooth or wrinkled colony phenotype. Unlike *C. albicans* and *C. tropicalis, C. parapsilosis* exists in the yeast and pseudohyphal form forming crepe-like and concentric patterns with no true hyphal extensions. Although all members of the *C. parapsilosis* species complex have diploid genomes, *C. parapsilosis* isolates are highly homozygous, while *C. orthopsilosis* and *C. metapsilosis* isolates exhibit extreme heterozygosity. These heterozygous characteristics likely stem from multiple hybridization events between closely related parents. A significant proportion of *C. metapsilosis* isolates have heterozygosity in the mating type-like locus, with introgression occurring at the *MTLa* locus. Mutations in the *ERG11* gene or its overexpression are linked to azole resistance in *Candida* species [6, 7,8,9,10]. Resistance to drugs has also been attributed to an increase in cell wall thickness, particularly an upregulation of cell wall chitin biosynthesis, rendering the cell impermeable to the drug. *C. parapsilosis* complex was divided into III groups, group 1 refers to the *parapsilosis* species, while group II and group II later became known as *orthopsilosis* and *metapsilosis;* the DNA sequence of *ITS1* sequences showed differences between the three groups.

The purpose of this study is to characterize nine *C. parapsilosis* Lebanese hospital isolates phenotypically and genotypically. Phenotypic characterization will be achieved through a battery of tests that target pathogenicity attributes such as virulence in a mouse model of disseminated candidosis, organ burden load, cell surface disruption, adhesion potential, ergosterol and biofilm formation. Genotypic analysis will be done through whole genome sequencing to identify documented and novel SNPs and mutations in key virulence and resistance genes. Phylogenetic comparison of isolates will also be done to analyze strain relatedness and clonality. The purpose is to attempt a correlation between the observed phenotypes and their specific genotypes.

## Materials and Methods

### *C. parapsilosis* strains

Nine *C. parapsilosis* hospital isolates were obtained from the Lebanese American University Medical Center, Rizk Hospital, Beirut in Lebanon. Species identification and broth microdilution antifungal susceptibility testing (Table 1) was performed by Husni et al. [11]. Four out of nine isolates were resistant to fluconazole, designated as isolates R1, R2, R3 and R4 and the five others designated as isolates S1, Ca, Co, S2 and Cm were sensitive to fluconazole. *C. parapsilosis* CDC317 MYA-4646 was used as a reference strain (GenBank accession no. GCF_000182765.1).

### *C. parapsilosis* culture

*C. parapsilosis* isolates were inoculated in 5mL potato dextrose broth (PDB) (Conda Laboratories) then incubated at 30°C overnight with shaking 90 rpm overnight. Finally, isolates were streaked onto potato dextrose agar (PDA) and incubated at 30°C for 48h. One colony was inoculated in fresh PDB for biofilm quantification, DNA extraction, cell surface disruption, organ burden load, and virulence.

### DNA extraction and whole genome sequencing

DNA was extracted using the “Quick-DNA Fungal/Bacterial Miniprep” kit (Zymo Research), following the manufacturer’s instructions. Elution was carried out using the BE buffer from the “NucleoSpin^®^ DNA Yeast” kit by Macherey-Nagel. Quantity and quality of DNA were assessed using a NanoDrop spectrophotometer. Library preparation, Illumina sequencing at a depth of 90X coverage, adapter trimming, and genome assembly were conducted by MicrobesNG in Birmingham, UK. Using the ATCC CDC317 MYA 4646 as a reference strain, variant calling was performed by MicrobesNG to predict variants relative to their reference. Sequences were deposited in NCBI through BioProject ID number PRJNA1091179.

### Sequence analysis and variant calling

Our sequence analysis approach consisted of scanning the genome for mutations in genes potentially involved in the development of azole resistance, biofilm formation and virulence. In total, we analyzed 96 genes (Supplementary Table 1). The genes of interest were translated using ExPASy translate tool (Swiss Institute of Bioinformatics) and the protein sequences were aligned using the Clustal Omega Multiple Sequence Alignment Tool (European Molecular Biology Laboratories, European Bioinformatics Institute) in order to determine amino acid substitutions in hospital isolates compared to the control strain. Mutations were verified by variant calling results performed by MicrobesNG. A literature search was performed in order to identify amino acid substitutions that have been previously documented.

### Single Nucleotide Polymorphism (SNP) Detection

We conducted a SNP analysis within the 8 genomes of the *C. parapsilosis* complex, comparing them to the SNPs present in the initial references’ strains. We utilized the snippy multicommand (snippy-base application v4.5.0) developed by Seemann [12] to generate a multiple alignment of the core genome against a common reference, which was CDC317 MYA-4646 for the *C. parapsilosis* isolates and Co 90–125 for *C. orthopsilosis* and BP57 for *C. metapsilosis*. This reference genome was chosen as the basis for comparison. The pipeline was employed to identify and document the variants, generating individual files for each isolate listing the specific variations.

### Annotation

Annotation of the reference genomes was achieved by using the following pipeline. First, gene prediction is done by using the combination of GeneMark-ES v4.71 and AUGUSTUS v3.4.0 by running BRAKER2 v2.1.6 with the fungus this step will identify the locus, CDS, and mRNA of the corresponding genes [13, 14,15,16,17,18,19,20]. Functional annotation was added by running the gff3 annotation file of the identified genes using Interproscan 5.50–84.0 [21] on the COG database, resulting in an XML file which in turn will be incorporated into the functional annotation pipeline Funannotate v1.8.7 [22,23,24]. The first step of this functional annotation is scanning the PFAM database using HMMscan default parameters (HMMer v3.3) (hmmer.org) and based on eggnog orthology data results using emapper v2.1.2 [25,26]. Furthermore, Diamond Blastp [27] used the UniProt DB v2023_02 and MEROPS v12.0 databases to generate separate functional annotation files that were later combined using Gene2Product v1.69. And finally, using Signalp v5.0 [28], secreted proteins were predicted.

### Phylogenetic analysis and ITS sequencing

To determine the phylogenetic relationship between the isolates, the core genome sequence, recombination data, and single nucleotide polymorphisms (SNPs) in conjunction with parsnp v1.2, a tool available in the Harvest suite was utilized. The corresponding reference genome was used for this analysis. SNPs identified in local collinear blocks were then employed to construct an approximate maximum-likelihood tree using FastTree, incorporating the general time reversible (GTR) model of nucleotide substitution. We applied the Shimodaira–Hasegawa test, implemented in FastTree2, to evaluate the support for significant clustering observed in the phylogenetic tree. For graphical representation and annotation, we utilized the interactive tree of life (iTOL) [29,30]. The sequence of the Internal Transcribed Spacer region ITS1 near the 5.8 rRNA gene of isolate “Cm” was Blasted onto the NCBI database and compared with various *C*. genome ITS sequences to determine phylogeny.

### Biofilm Assay

The protocol of biofilm biomass quantification was adopted from [6,31] with slight modifications. The culture medium adopted was PDB and OD measurements were taken at 590 nm. This experiment was performed in biological and technical triplicates and ATCC CDC317 MYA 4646 was used as a control reference.

### Ergosterol

To extract plasma membrane ergosterol we used the protocol described by Arthington-Skaggs et al. [32]. Optical densities (ODs) were measured following the protocol detailed in Fattouh et al. [7] and these OD values were used to calculate ergosterol content by relying on the formulas in [7,32]. This experiment was performed in biological triplicates and CDC317 MYA 4646 and the control strain.

### Sodium Dodecyl Sulfate (SDS) Susceptibility Surface Disruption Assay

Susceptibility to SDS was determined by microdilution; exponentially grown cultures were diluted to 10^6^ cells/mL, and ten-fold serial dilutions were done. 5 μL of each dilution was spotted on PDA plates and incubated at 30°C on 0.025% SDS plates, and plates lacking SDS (control). All isolates were spotted in triplicates and were photographed at 24h 48h and 72h [6].

### Virulence Assay and Organ Fungal Burden Load

To induce a disseminated systemic infection, the Fattouh et al. [7] protocol was adopted. 6 weeks old female BALB/c mice were injected via their tail vein with 5×10^8^ cells in 200 μL 1X Phosphate Buffered Saline solution (PBS) to generate a systemic infection. 6 mice were injected per isolate, and a group of 6 mice were injected with 1X PBS as a control [6,7,33,34]. Mice manipulation followed all ethical standards of the Lebanese American University Institutional Care and Use Committee which approved the execution of this experiment in June 2022 under approval code LAU.ACUC.SAS.RK5.12/June/2022. For the organ burden load experiment, 6 weeks old female BALB/c mice were injected with 5×10^7^ cells in 200 μL PBS via the tail vein to induce a systemic infection. Two mice were injected per isolate, and 2 mice were injected with 1X PBS as a control [34]. On day 4, mice were euthanized using carbon dioxide inhalation and designated organs (liver and kidneys) were taken and preserved in 5mL 1X PBS. Organs were homogenized using a Rotor Stator Homogenizer, 1 mL were taken from each homogenized organ and spread on a PDA plate. Plates were incubated at 30°C and colonies were counted after 48h [35].

### Statistical analysis

Tables were generated using Microsoft Excel. Figures as well as all statistical analyses were executed using GraphPad Prism versions 7.00 or 10.1.2. For the virulence assay, the Mantel-Cox test was performed. Furthermore, for the quantification of fungal load, biofilm, and ergosterol, Kruskal Wallis test and Dunn’s multiple comparisons test were performed. Any p-value below or equal to 0.05 was considered significant.

## Results

### Species Identification and Phylogenetic analysis

**Figure 1:**
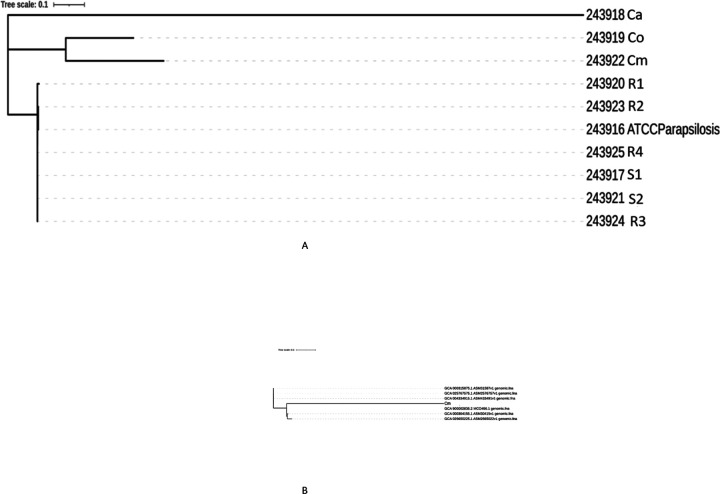
**a: SNP based phylogenetic tree of hospital isolates.** *C. parapsilosis* isolates R1, R2, R3, R4, S1 and S2 cluster together and are phylogenetically close as opposed to Co and Cm that cluster together separately. Isolate Ca is phylogenetically divergent from all the other isolates. **b: SNP based phylogenetic tree of Cm with 6 *C. orthopsilosis* genomes.** Cm is significantly divergent from all *C. orthopsilosis* reference sequences, suggesting it might belong to a different species.

Based on the MicrobesNG whole genome sequencing data, strains Co and Cm were deemed to be C. parapsilosis and Ca *C. albicans*. As a non *parapsilosis* complex species, The Ca strain was subsequently removed from all further phenotypic and genotypic analysis. A SNP based phylogenetic tree was generated for the sequenced samples and the reference genome (CDC317 MYA-4646). The tree showed that isolates Ca, Co and Cm were distinct from the rest with significant sequence divergence, whilst the other 6 isolates and the reference strain clustered together with high sequence similarity (Fig. 1 a). However, for two isolates Cm and Co, the variant calling did not match the *C. parapsilosis* CDC317-MYA4646 reference as the sequence exhibited many mutations and unmatched ORFs, so we suspected that they might belong to a different species. The Cm and Co sequences were blasted on NCBI, and Co exhibited very high sequence similarity to *C. orthopsilosis*. The remaining isolate, Cm did not match with the *C. orthopsilosis* (Co_90–125) reference sequence from NCBI exhibiting many mutations, and indels upon variant calling. To support this, a phylogenetic tree was generated for Cm alongside six *C. orthopsilosis* reference genomes (Fig. 1b). The phylogenetics tree showed that Cm is significantly divergent from all C. orthopsilosis reference strains, further justifying our suspicions that it is a different species. Finally, the ITS1 region of Cm was blasted against *Candida* ITS1 regions. The highest sequence similarity was for *C. metapsilosis* ITS1. As such the Cm isolate is *C. metapsilosis*.

### Mutational analysis

Whole genome sequencing was performed on all 8 isolates, followed by variant calling. For the C. *parapsilosis* isolates, mutational analysis on the genes in Table S1 was performed. For the *orthopsilosis* and *metapsilosis* isolates, the mutational analysis is shown in Tables S2 and S3. Note the high clonality amongst *C. parapsilosis* isolates whereby similar mutations are observed in different strains.

### Biofilm Assay

**Figure 2:**
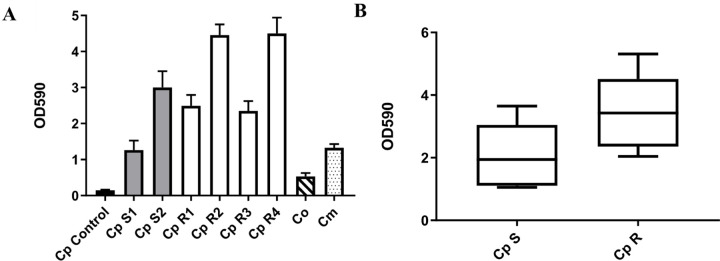
Biofilm formation. **(A)**Biofilm formation is represented as measurements of optical density at 590 nm (OD590) for all *C. parapsilosiscomplex* isolates. The black bar graph represents the *C. parapsilosisreference* CDC317 MYA-4646, control, while the gray bar represents the fluconazole sensitive *C. parapsilosis* isolates, and the white bar graphs represent the fluconazole resistant *C. parapsilosis* isolates. The hatched bar is for C. orthopsilosis, and the dotted one is for *C. metapsilosis*. For all isolates and the control, quantification of biofilm amounts was performed in biological triplicates. Mann-Whitney test: Cp-S vs. Cp-R: p = 0.0055 is significant, Cp-S vs. Co: p = 0.0001 is significant, Cp-S vs. Cm: p = 0.2908 is not significant, Cp-R vs. Co: p < 0.0001 is significant, Cp-R vs. Cm: p < 0.0001 is significant, and Co vs. Cm: p = 0.0022 is significant. **(B) Kruskal Wallis test.** Boxplot graph was generated to compare the potential of biofilm formation in fluconazole-resistant clinical C. parapsilosis isolate groups (R1, R2, R3, R4) with that of the reference CDC317 MYA-4646 and the fluconazole-susceptible isolates group (S1, S2). Resistant isolates exhibited more potential of biofilm formation than the sensitive strains. For all isolates, the experiment was performed in biological triplicates. p < 0.0001 for Cp sensitive isolates compared to control is significant, p < 0.0001 for Cp resistant isolates compared to control is significant.

All isolates exhibited an increase in biofilm formation compared to the control strain (Fig. 2A). However resistant isolates exhibited more biofilm formation on average compared to sensitive strains (Fig. 2B). Fluconazole resistant isolates (R1, R2, R3, R4) revealed an increase in biofilm formation compared to sensitive strains (S1, S2) with (x ~1.75) fold increase. Fluconazole sensitive isolates showed (x ~ 2.13) folds increase in the potential of biofilm formation when compared to the reference CDC317 MYA-4646, while resistant isolates exhibited a (x ~ 4.5) increase in biofilm formation compared to the control. Interestingly, both Co and Cm showed less biofilm formation than sensitive and resistant *C. parapsilosis* isolates.

### Ergosterol Assay

One of the main mechanisms involved in drug resistance in *Candida* species is the upregulation of ergosterol. When ergosterol deposition increases, the plasma membrane becomes thicker blocking fluconazole from entering the cell, resulting in resistance. We thus measured ergosterol content in both our susceptible and resistant isolates. Although we observed an increase in ergosterol content in some of the sensitive and resistant isolates, no correlation was found between drug resistance and ergosterol content. Isolate Cp S1 showed 92% increase in ergosterol content compared to Cp S2 that exhibited a decrease in approximately 102% of its ergosterol content compared to the control. On the other hand, Cp R1, Cp R3 and Cp R4 showed a decrease in ergosterol content compared to the control, while Cp R2 showed an ergosterol increase of 43%. Our data suggests that ergosterol content might not be the only mechanism to achieve resistance in our isolates. Based on the Mann-Whitney test, observed differences were not statistically significant even though a slight increase in ergosterol content in sensitive *parapsilosis* isolates was found.

### Virulence Assay

**Figure 3:**
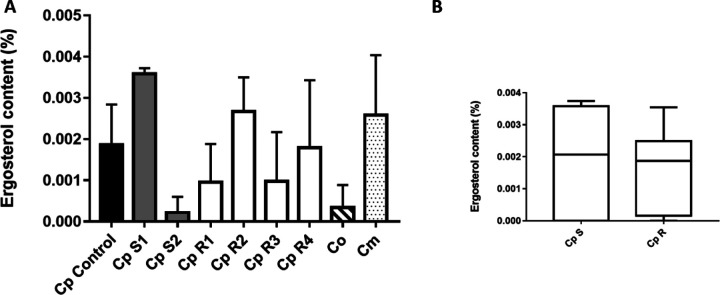
Ergosterol content. **(A)** In the Mann-Whitney test, the black bar graph represents the C. parapsilosis reference CDC317 MYA-4646, control, while the gray bar represents the fluconazole sensitive C. parapsilosis isolates, and the white bar graphs represent the fluconazole resistant C. parapsilosis isolates. The hatched bar is for C. orthopsilosis, and the dotted one is for C. metapsilosis. For all isolates and the control, quantification of ergosterol amounts were performed in biological triplicates. Cp-S vs. Cp-R: p = 0.6786 is not significant; Cp-S vs. Co: p = 0.5238 is not significant; Cp-S vs. Cm: p = 0.5 is not significant; Cp-R vs. Co: p = 0.1824 is not significant; Cp-R vs. Cm: p = 0.2901 is not significant; Co vs. Cm: p = 0.1 is not significant. **(B)** In the Kruskal Wallis test & Dunn’s multiple comparisons test, the boxplot graph was generated to represent the ergosterol content in fluconazole sensitive and fluconazole resistant clinical C. parapsilosis isolate groups and upon comparison with the control, CDC317 MYA-4646. p < 0.0001 for Cp sensitive isolates (S1 and S2) compared to control (significant); p < 0.0001 for Cp resistant isolates (R1, R2, R3, R4) compared to control is significant too. For all isolates, the experiment was performed in biological triplicates.

Each isolate was injected into the tail vein of 4 mice in a disseminated model of infection. One out of 4 mice injected with the sensitive isolate S1 was moribund by day 2, whereas no death was observed in isolates S2, Co, Cm, R1, R2, R3 and R4 (Fig. 3). Overall, our data analysis shows that fluconazole resistance is associated with a slight attenuation of virulence in the *parapsilosis* complex as one mouse injected with S1 and one mouse injected with the sensitive *parapsilosis* ATCC control strain died, but none of the mice injected with resistant strains were moribund.

### Organ fungal burden Load

**Figure 4:**
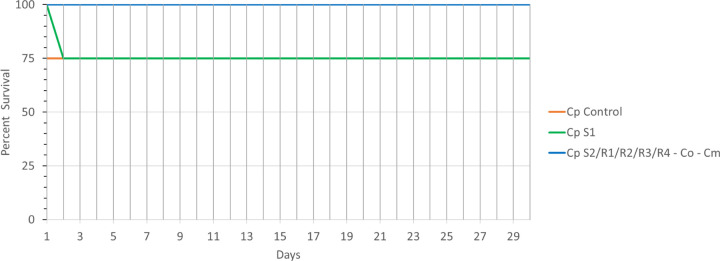
Figure 3: Murine Model Assay. Virulence potential in a murine disseminated model of infection. BALB/c mice were injected with 10^8^ cells of CDC317 MYA-4646 and fluconazole-sensitive and resistant isolates and monitored for survival over 30 days. The survival rate in most fluconazole-susceptible and all resistant isolates was 100%. Mantel-Cox test (log-rank test): p = 0.5366 (not significant). Logrank test for trend: P = 0.0552 (not significant).

Since no significant differences were observed in our virulence assay, we decided to assess fungal burden load for each of the isolates to determine whether discrepancies amongst isolates are observed. However, no significant difference was observed between sensitive and resistant isolates as far as organ burden load. All isolates exhibited an increased burden load in the kidneys compared to the liver (Fig. 4) with a significant p value of 0.0024. On the other hand, fungal burden load in kidneys and liver for sensitive versus resistant strains compared with the control was not significant with a p value > 0.9. We did we observe any significant differences in organ burden load within the three species.

### Cell Wall Disruption

**Figure 5:**
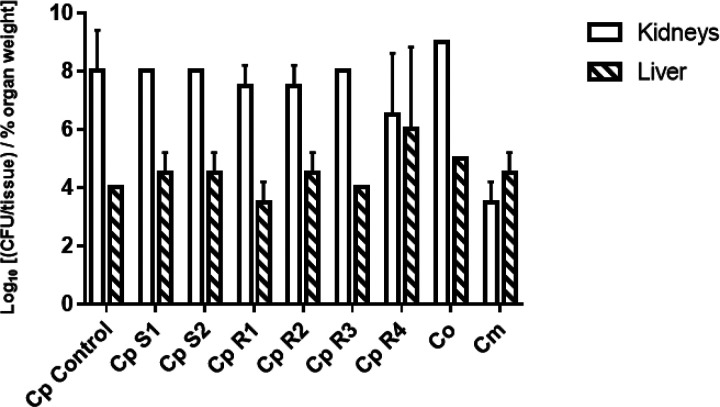
Figure 4: Fungal Burden Load. BALB/c mice were intravenously injected in the tail vein with 10^8^ cells for each of the indicated strains (S1, S2, R1, R2, R3, R4, Co, Cm). Data are expressed as the log10 colony-forming units (CFUs)/tissue of *%* organ weigh from kidneys and liver homogenates at day 4 after the challenge. The log10 CFUs from both kidneys were combined and averaged. Log10 counts were compared for statistical significance by Mann-Whitney test with a p = 0.0024 (significant). A p-value <0.05 was considered to be statistically significant. Moreover, *C. parapsilosis* sensitive vs. *C. parapsilosis* resistant kidneys fungal load has a p = 0.4182 (not significant), for the liver fungal load p = 0. 5899 (not significant). *C. parapsilosis* sensitive vs. *C. orthopsilosis* kidneys fungal load have a p = 0.0667 (not significant), for the liver fungal load p > 0.9999 (not significant). *C. parapsilosis* sensitive vs. *C. metapsilosis* kidneys fungal load has a p = 0.0667 (not significant), for the liver fungal load p = 0.4667 (not significant). *C. parapsilosis* resistant vs. *C. orthopsilosis* kidneys fungal load has a p= 0.0222 (significant), for the liver p = 0.9333 (not significant). *C. parapsilosis* resistant vs. *C. metapsilosis* kidneys fungal load has a p = 0.222 (significant), for the Liver p = 0.2444 (not significant). *C. orthopsilosis* vs. *C. metapsilosis* for the kidneys fungal load is p = 0.3333 (not significant), and for the liver p > 0.9999 (not significant).

The cell wall is a crucial component of fungal cells that plays a critical role in maintaining cell shape, protection from osmotic stress, and the host. Sodium Dodecyl Sulfate (SDS) is a detergent that can disrupt the integrity of the fungal cell wall by solubilizing its components, such as glucans, chitin, and proteins. This disruption can cause fungal cells to become more susceptible to lysis, stress, and other environmental insults. Strains were 10x serially diluted and grown in various concentrations of SDS. No growth was observed at concentrations above 0.025% SDS. Fluconazole resistant isolate R3 was significantly more resistant to SDS than the control strain, while fluconazole sensitive strains S1, S2, and Cm were less resistant to SDS% (Fig. 5). Decreased growth in the sensitive strains implies a relatively weak and permeable cell wall, which can be easily disrupted by SDS. In contrast, resistant fungal strains may have a thicker, more complex, or more impermeable cell wall that is less susceptible.

## Discussion

In Lebanon, as well as on a global level, there has been limited research conducted on the infections of the fungal pathogen *C. parapsilosis* and its emerging azole resistance in hospital isolates. Most studies focus on *C. albicans* as it is the most commonly isolated *Candida* pathogen [8]. Furthermore, there has been no research as to the relationship between resistance patterns and pathogenic attributes of this yeast species. As such, the primary objective of this research was to characterize our hospital isolates, both from a phenotypic and genotypic perspective. Phenotypic characterization entailed a series of tests targeting pathogenesis traits, such as virulence in a mouse model of disseminated candidiasis, organ burden load, biofilm formation, ergosterol content, and cell wall disruption. Genotypic analysis was conducted through whole genome sequencing and SNPs identification to identify known and previously uncharacterized mutations in critical virulence and resistance genes. SNP analysis was done through 3 different methods MicrobesNG, manually through EXPASY and Clustal Omega Multiple Sequence Alignment Tool, and through snippy multicommand, to confirm our results. Additionally, we performed a phylogenetic analysis of the isolates to examine their relatedness and clonality. The overarching goal is to establish a relationship between the observed phenotypes, resistance profiles, and specific genotypes.

Lanosterol 14-demethylase, part of the cytochrome P450 enzyme family, plays a crucial role in ergosterol synthesis. The *ERG11 (CPAR2_303740)* gene encodes this enzyme, and it serves as a target for azole medications. Azoles, with fluconazole being a prominent example, are frequently employed in the management of candidiasis. *ERG11* mutations are known to be associated with azole resistance, and within azole-resistant strains, the Y132F substitution in *ERG11* is the sole substitution observed in the literature [36]. Moreover, in a study on azole resistance mechanisms in *C. parapsilosis* by Grossman et al. [37], the sequences of *ERG11* were examined and the Y132F substitution was found in 56.7% of their fluconazole-resistant isolates. They concluded that this mutation likely contributes significantly to fluconazole resistance in *C. parapsilosis*. Berkow et al. [38] also found this mutation, in addition to another substitution, R398I. The R398I substitution was present as the sole mutation in susceptible isolates, or alongside the Y132F substitution. Our findings are compatible with their data as R398I was detected in sensitive isolates S1 and S2, and the F132Y detected in resistant isolates R1, R2, R3, and R4. Furthermore, previous research showed that mutations in *ERG3* and *ERG6* are also associated with azole resistance, [39]. Stefanek et al. [40] observed two mutations S208G and S304G in the *ERG6* gene involved in azole resistance; these two mutations are present in all our *C. parapsilosis* resistant isolates, but even in our sensitive isolates suggesting that other mechanisms might be responsible for resistance. One other mechanism of drug resistance is the upregulation of efflux pumps; these efflux pumps pump the drug out of the intracellular membrane. *CDR1 (CPAR2_405290)* is one of the major efflux pumps involved in fluconazole resistance; *CDR1* serves as a versatile ATP-Binding cassette (ABC) efflux transporter, responsible for pumping out and expelling azoles from the cell [10]. This action decreases the intracellular drug accumulation, ultimately leading to the development of resistance. In our isolates, *CDR1* was mutated in all the resistant isolates R1 (I1287V, I969T), R2 (N1132D), R3 (I1287V) and R4 (I1287V) possibly leading to drug resistance. Interestingly we did not observe a upregulation of ergosterol content in our isolates, which lends further credibility to the fact that pumping out the drug might be a more plausible mechanism of resistance in our isolates.

The formation of biofilm in microorganisms is regarded as both a characteristic of pathogenicity and a protective response to unfavorable conditions, such as fluctuations in pH, temperature, nutrient availability, or exposure to antifungal drugs. *Candida* accomplishes this by generating a dense network of extracellular polymeric substances, which acts as a protective barrier against external stresses and provides an environment conducive for growth and survival. This increased concentration of the matrix, compared to planktonic cells, offers greater protection against drugs and results in enhanced resistance. *FAS2 (CPAR2_807400)* was found to be essential for the proper development of biofilms with absence of *FAS2* led to reduced virulence in a systemic mouse infection model [41]. CFEM (Common in Fungal Extracellular Membranes) proteins were associated with abnormal biofilm formation and decrease in virulence. Interestingly S1, S2, R1, R2, R3 and R4 all have the same T99A mutation in *FAS2* and all have attenuated virulence. In addition, strains S1 and S2 have a mutation in *CPAR2_407410 (MP65)* which is known to be involved in biofilm formation; mutated forms of the gene are known to produce less biofilm [42,43]. Taff et al. [44] showed that azole susceptible strains produce less biofilm; S1 and S2 in our study produce less biofilm in accordance with the literature. We also examined *EFG1,* which is a gene associated with biofilm formation, and it turned out to be mutated, which might cause abnormal biofilm formation [45].

Lockhart et al. [46], reported that the combined prevalence of *C. orthopsilosis* and *C. metapsilosis* in *C. parapsilosis* complex infections is generally less than 10%. In our case, they account for 25% of our samples. However, it should be noted that one reason for this could be the low sample size in our study. Lockhart et al. [46] also noted that *C. orthopsilosis* and *C. metapsilosis* isolates tend to remain susceptible to fluconazole. In our study, we found that *C. metapsilosis* (Cm) and *C, orthopsilosis* (Co) exhibited susceptibility to fluconazole, with MIC values of 0.5 and 1, respectively, in accordance with their study. In addition to the susceptible *C. metapsilosis* and *C. orthopsilosis* isolates, *C. parapsilosis* resistant and sensitive isolates did not exhibit strong virulence in murine disseminated infections which is correlated to previous studies that show no correlation between drug resistance and virulence [9].

As far as *orthopsilosis,* we analyzed *ERG11* and found multiple mutations such as Q211K, A421V, and V485I in isolates that retained susceptibility to fluconazole. This aligns with the findings of Xiang et al. [47]. Additionally, we observed mutations in other *ERGs,* but these mutations were not previously documented in the literature. In our study, we discovered mutations in the *MDR1* and *MRR1* efflux pumps in *C. orthopsilosis* (Co). However, we did not find any prior literature that specifically mentioned these mutations (supplementary table). Mutations in the *CHS3* gene affect chitin deposition and virulence [48]. These mutations may be responsible for the observed reduction in virulence in Co strains with the following mutations.

Significant heterozygosity and diversity are prominent features of the genetic landscape of *C. orthopsilosis* and *C. metapsilosis,* which could have been influenced by hybridization events. The shortage of ATCC references, resulting in a lack of well-characterized strains for comparison, further complicates mutational analysis. However, our study confirms the high rate of diversity found in these isolates which is a hallmark of *orthopsilosis* and *metapsilosis* as they are the result of hybridization events which can be seen from the vast number of mutations documented in the literature[3,49], and observed in tables S2 and S3 in our isolates. It is widely thought that *orthopsilosis* is an ancestor to *parapsilosis,* justifying why there is more sequence diversity in *orthopsilosis* versus *parapsilosis,* that is a “newer” species and that has not yet had enough time to diverge significantly [3].

One interesting but unexpected aspect of our study is the rate of isolate-misidentification 50. All our isolates were labeled by our tertiary care center as *C. parapsilosis* [11]. However, based on our whole genome sequencing data a significant percentage of our isolates- 3 out of 9- were misidentified. For two of the isolates this misidentification might not have significant clinical implications since *C. orthopsilosis* and *C. metapsilosis* are part of the same family as *parapsilosis* and treatment is very similar for all three [48]. However, misidentification as *C. albicans* is more serious and can have adverse effects on treatment, and patient wellbeing. This stresses the need of using DNA sequencing data for proper microorganism identification.

Our study is based on 9 isolates, a relatively low sample size as far as statistical analysis is concerned. The reason for this is that the rate of *C. parapsilosis* hospital infection is low to begin with compared to *C. albicans* or bacterial pathogens for example. The fact that a third of them turned out to be non *parapsilosis Candida* affected our ability to determine statistically significant correlations between resistant and sensitive isolates.

An interesting result from our study is the high clonality and sequence similarity amongst our *C. parapsilosis* isolates. This can be seen in the phylogenetic tree analysis whereby all *C. parapsilosis* isolates cluster tightly together with a very low rate of substitutions amongst them as can be seen through the very short branch lengths that typically represent nucleotide substitutions per site. As opposed to the high rate of substitutions within *orthopsilosis* and *metapsilosis* isolates we observed a relatively low mutation rate and diversity within our *parapsilosis* isolates, similar to previous findings. The genes *FAS2, MDR1, ALS6, ALS11, CPAR2_700020, SAP7, HWP1, UPA* and *UME6* had mutations which were exclusively found in resistant isolates and mutations in *CPAR2_302400* and *CPAR2_110220* found in the sensitive ones. This high rate of clonality is supported by our mutation analysis whereby similar mutations appear in multiple isolates. Utilizing mutation analysis to unveil the genetic basis of resistance represents a critical initial step in enhancing our understanding of *Candida* pathophysiology and in devising more potent strategies for combating drug resistance. Isolates with high sequence similarity and similar mutations isolated from the same ward at the same time, which is the case for our isolates, is an indication of a hospital outbreak. However, the lack of hospital data and patient history prevents us from further analysis and conclusions.

In conclusion, this is the first study of its kind to address *C. parapsilosis* complex isolates from both a phenotypic and genomic perspective. Our data has revealed variations in both the physical characteristics and genetic makeup of azole-resistant and azole-sensitive isolates. While our *parapsilosis* isolates displayed largely similar physical traits, the key distinction was observed in their ability to form biofilms, with the resistant isolates showing a higher propensity for enhanced biofilm formation. Our study also revealed hospital misidentification rates. Finally, it is well known that in addition to genomic mutations, drug resistance can be caused by upregulation of expression. Future work entailing a larger set of isolates with patient history, coupled with a transcriptomic approach would be of interest.

## Figures and Tables

**Figure 6:**
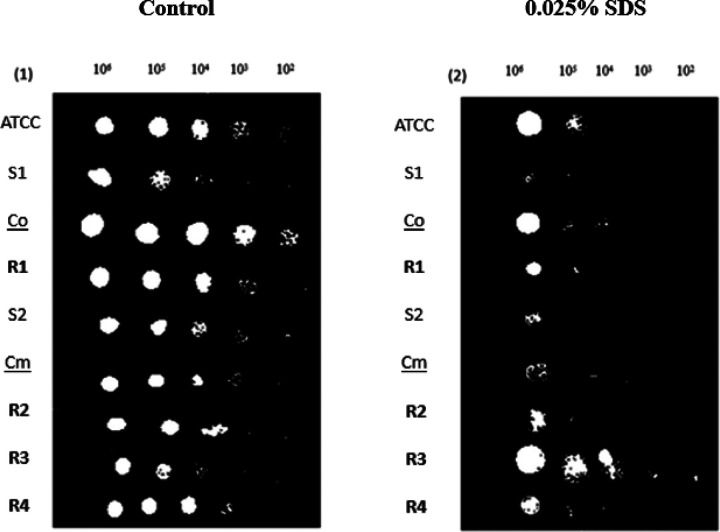
Figure 5: Cell Surface Disruption Assay. Strains were 10X serially diluted and plated on media containing 0.025% SDS. Colony formation was photographed after 24h. **(1)** Shows the control plates with no SDS added. **(2)** Shows the growth of sensitive and resistant strains with the presence of SDS. R3 showed more resistance to SDS and more survival potential than the other resistant isolates, followed by R4 and R2; for the sensitive isolates, the S1 and S2 were susceptible to SDS, and their cell wall was being disrupted.

**Table 1:** Broth Microdilution Results. Isolate R1, R2, R3 and R4 are resistant to fluconazole, whereas isolate S1, Ca, Co, S2 and Cm were sensitive.

Isolates	Fluconazole (MIC) \varvec*μ*\varvec*g*/\varvec*m*\varvec*L*	
**S1**	0.5	Sensitive
**Ca**	1	Sensitive
**Co**	1	Sensitive
**R1**	32	Resistant
**S2**	2	Sensitive
**Cm**	0.5	Sensitive
**R2**	8	Resistant
**R3**	8	Resistant
**R4**	16	Resistant

**Table 2:** List of *C. parapsilosis* mutations

Genes	Roles	isolates	Protein Mutations
**FAS2; CPAR2_807400**	Virulence	S1, S2	A328V; I330T; S1349N
	Biofilm Formation	R1, R3, R4	I330T; S1349N
**CPAR2_110220**	Resistance to SRS	S1	P441T; R478K
	Virulence		
	Resistance to caspofungin		
	Resistance to fluconazole		
**ERG11, CPAR2_303740**	Resistance to azole	S1, S2	F132Y; R398I
	Ergosterol	R1, R2, R3, R4	F132Y
**FKS1, CPAR2_106400**	Resistance to caspofungin	S2	A1422G; M1499I
	Biofilm Formation		
**UPC2, CPAR2_207280**	Resistance to ketoconazole	R1	N455R
**CFEM4, CPAR2_402890**	Biofilm formation	S1, S2, R1, R3, R4	T99A
	Virulence		
**ATP6; CPAR2_203290**		S1, S2	A120T
		R1	T191A
**CPAR2_207540**	Resistance to fluconazole	R3, R4	I489V
	Virulence		
**CDR1; CRAR2_405290**	Resistance to fluconazole	R1	I1287V, I969T
		R2	N1132D
		R3, R4	I1287V
**ERG4; CPAR2_502980**	Virulence	R1, R3	P63S
	Ergosterol	R3	P63S
**ERG6; CPAR2_405010**	Biofilm formation	S1, S2, R1, R2, R3, R4	S208G; S304G
	Virulence		
	Ergosterol		
**UPC2; CPAR2_207280**	Resistance to ketoconazole	R1	N455D
	Ergosterol		
**STP4; CPAR2_211740**	Resistance to caspofungin	S1, S2, R1, R3, R4	L195P
	Resistance to SDS		
**CHS1; CPAR2_805640**	Virulence	R1	P52L
**MP65; CPAR2_407410**	Adhesion	S1, S2	F369L
	Biofilm formation		
	Virulence		
**SAPP3; CPAR2_102420**	Virulence increased	R1, R3, R4	T359I
**GCN4; CPAR2_806570**	Biofilm formation: decreased	S2	G120D
**NTC1; CPAR2_803760**	Resistance to caspofungin	S1, S2, R1, R3, R4	L671Q
**CHS3; CPAR2_801800**	Virulence	S1, S2, R1, R3, R4	L38R
**ALS6; CPAR2_404790**	Adhesion	R1	S2190T
		R3, R4	I1430T, S2190T
**ALS7; CPAR2_404800**	Virulence	R2, R3, R4	E765D
**ALS11; CPAR2_404780**	Virulence	S1, S2	G94E, L350V
		R1, R3, R4	L350V, G1000R
**CFEM5; CPAR2_300110**	Biofilm Abnormal	R1, R3	S224T
		R4	G807S, P805L, A803T, G797S, L735P, T733A, G717S, P715L, S224T, P795L, A793T, A783T, S737G
**CPAR2_700020**		S1, S2	V171I
**CDRV CPAR2_700030**	Resistance to fluconazole	R1	L591I
		R3, R4	D127Y
**CPAR2_700100**	Virulence	S1, S2, R1,R3, R4	F46I
**CPAR2_700140**	Resistance to caspofungin	R1, R3, R4	N140S
**EFG1; CPAR2_701620**	Resistance to SDS	S1, S2, R1, R3, R4	E452G
	Resistance to caspofungin		
	Virulence		
	Biofilm Formation		
**SAP7; CPAR2_105640**	Virulence decreased	R1, R3, R4	Q66H
**SAP9; CPAR2_102610**	Virulence decreased	R1, R3, R4	S439G
**CPAR2_302400**		R3, R4	I114M
**ERG25; CPAR2_801410**	Resistance to fluconazole	R3	D9N
	Ergosterol		
**HWP1; CPAR2_403520**	Biofilm formation	S1, S2	L461S
	Virulence	R1, R4	G1129E, Q1128P, G1127R, E1126G, S1114G, L1113R P975S, S974G, L973P, L461S, P1115S
		R3	G1129E, Q1128P G1127R, E1126G, L461S
**EPA; FUN31; CPAR2_808450**	Resistance to caspofungin	R1, R3, R4	D396G
**EAP1; CPAR2_805000**	Resistance to SDS	R3	T663S
**UME6; CPAR2_803820**	Virulence	S1, S2	C547G, T46A
		R1, R3, R4	C547G
**FLO8; CPAR2_601080**	Biofilm formation	S1, S2, R1, R3, R4	G1052S
	Virulence		
